# Case reports: a variety of clinical presentations and long-term evolution of Bochdalek hernias

**DOI:** 10.3389/fsurg.2023.1150241

**Published:** 2023-05-26

**Authors:** Sebastien Frey, Maurice Chazal, Eric Sejor, Patrick Baque, Jerome Mouroux

**Affiliations:** ^1^ Department of General Surgical Emergency, Pasteur 2 Hospital, University Hospital of Nice, Nice, France; ^2^ Université Côte d’Azur, Nice, France; ^3^ Department of General Surgery, Princess Grace Hospital, Monaco, Monaco; ^4^ Department of Digestive Surgery and Liver Transplantation, Archet 2 Hospital, University Hospital of Nice, Nice, France; ^5^ Department of Thoracic Surgery, Pasteur 1 Hospital, University Hospital of Nice, Nice, France

**Keywords:** Bochdalek hernia, diaphragmatic hernia, congenital hernia, thoracic surgery, thoracotomy, abdominal surgery

## Abstract

Bochdalek hernias are the most common congenital diaphragmatic hernias, followed by Morgagni hernias. The failure of closure of the pleuroperitoneal membrane results in a posterolateral foramen, which can remain silent until adulthood. They remain a rare pathology with nearly a hundred cases published. Its clinical presentation is variable, making its diagnosis challenging for clinicians. Additionally, its symptoms are not necessarily representative of the content of the hernia. Its management is balanced between the abdominal and the thoracic approaches. However, no guidelines or algorithms are available to help surgeons in the decision-making process. We report here four consecutive cases of symptomatic Bochdalek hernias. Each case has a singular presentation, and we share how they were approached at our institution. In particular, this series shows no reoccurrence in 10+ years of follow-up in two cases and 20+ in one case, underlying the importance of surgical management when Bochdalek hernias are symptomatic.

## Introduction

Diaphragmatic hernias are a group of conditions acquired or congenital, common or rare, or asymptomatic or complicated. Bochdalek hernias (BH) are the most common congenital diaphragmatic hernias, due to the failure of closure of the pleuroperitoneal membrane resulting in a posterolateral foramen (1, 2). While it is most often diagnosed and treated during the perinatal period, it can remain undiagnosed until adulthood when asymptomatic. However, the prevalence of incidental BH among adults is lower than 0.17% of abdominal contrast-enhanced computed tomography (CT) (3). It is believed that a phase of intra-abdominal pressure precedes the symptomatic state. Nevertheless, a precipitating factor is only found in 25% of cases (4). The content of the hernia is exotic—including the bowel, liver, kidney, or omentum, and explains its various presentations: either due to a strangulation mechanism of the content or due to a compressive mechanism on the lungs. Numerous symptoms have been described and can concern the respiratory as well as the digestive area. Surgical management is required for symptomatic cases and might be emergent when a life-threatening condition is present. So far, no guidelines nor algorithms have been proposed for the management. We report here our experience over the last 20 years. Four cases of symptomatic Bochdalek hernias are presented in a chronological order. We discuss the variety of their presentations and the strategic choices made for their management.

## Case reports

### Case 1

**Figure 1:**
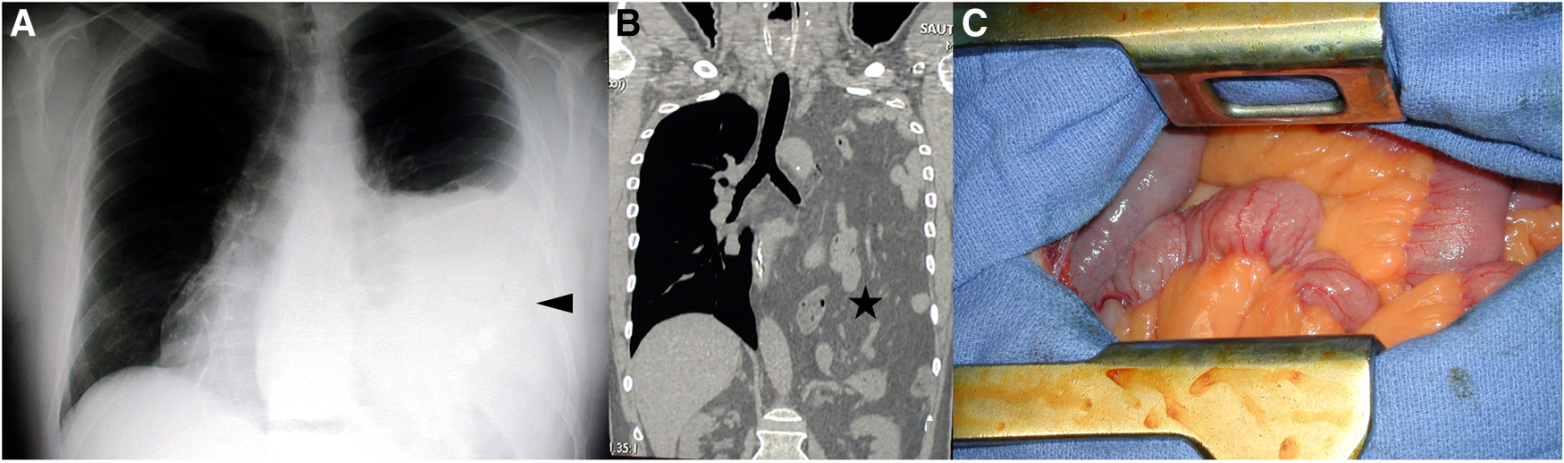
Case 1: (**A**) The thoracic x-ray is showing a left thoracic opacity up to the aortic arch; (**B**) the corresponding thoraco-abdominal CT in the frontal view showing the presence of intestinal content in the left thoracic cage; and (**C**) the herniated intestinal content following left thoracotomy.

In 2005, a 59-year-old male, with a history of tobacco consumption, presented at our emergency unit for acute dyspnea associated with intestinal obstruction syndrome. He complained of shortness of breath for the past 3 months. A thoracic x-ray found a right opacity up to the aortic arch (Figure 1). A thoraco-abdominal CT showed the presence of nearly the entire small bowel, the left colic angle with part of the transverse and left colon, and the spleen, herniated in the left thoracic cage (Figure 1). The stomach appeared distended. No history of trauma was found at interrogation. Medical management was first performed, including the placement of a nasogastric tube and the administration of antispasmodic medications. After the improvement of his clinical condition, a surgical procedure was planned 7 days following his admission. A left thoracotomy was first performed (Figure 1). The reduction was difficult, and to avoid twisting the intestine, a mid-laparotomy extended to a left phrenotomy was required. Many adhesions had to be taken down. Following the reduction of the content from the thoracic cavity, the left lung appeared crushed, but slow and repeated ventilation led to its expansion. The repair required the use of a polytetrafluoroethylene mesh (Gore-Tex®), attached posteriorly to the atrophic muscular bead and anteriorly to the mid-part of the diaphragm. An improvement of the FEV_1_ was noted, going from 2.46 L pre-operatively to 3.17 L postoperatively. The postoperative course was uneventful, and there was no evidence of recurrence over the last 22 years. During a recent hospital stay in pneumology, a CT confirmed these data.

### Case 2

**Figure 2:**
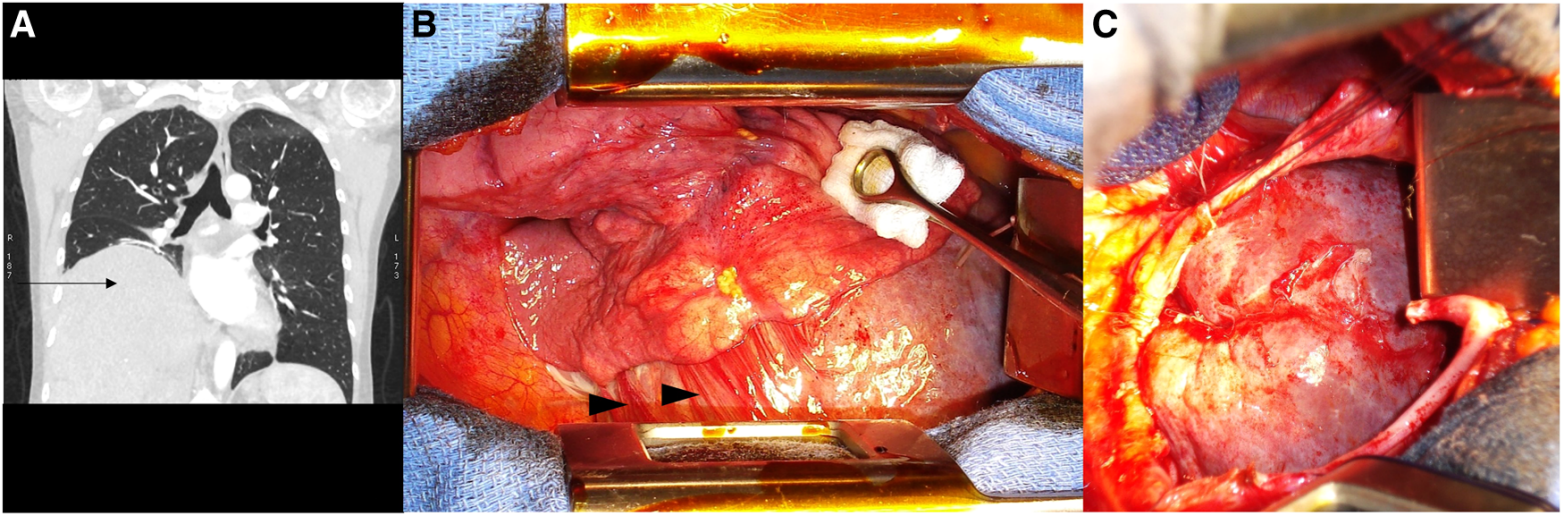
Case 2: (**A**) The pre-operative thoraco-abdominal CT in the frontal view is showing the liver protruding in the right thoracic cage (black arrow); (**B**) the per-operative view upon right thoracotomy with chronic adhesions (black arrowheads) between the lower right lobe, the liver, and the diaphragm; and (**C**) the per-operative view after reduction of the herniated liver and the diaphragmatic defect.

The second case is a 37-year-old female with a history of endometriosis and two pregnancies, but no history of trauma. In 2012, she had a laparoscopic left colectomy with left annexectomy for infiltrating endometriosis nodules. During the surgery, the surgeon noticed a right diaphragmatic dehiscence covered by the liver. The surgery was complicated with a left ureteral leak. A laparoscopic lavage, surgical drainage, and the insertion of a double-J ureteral catheter were performed. During the postoperative course, she complained of the inability to lie down on her left side, although she has chronic pelvic pain, related to endometriosis. A follow-up CT confirmed the diagnosis of right BH: the liver was protruding in the right thoracic cage, and a left pleural effusion was associated (Figure 2). The forced expiratory volume in 1 s (FEV_1_) was 30% lower than the predicted value. The tolerance of the clinical symptoms was sufficient to plan surgery after the resolution of the ureteral leak. At 3 months, and after the removal of the double-J ureteral catheter, a right thoracotomy in the eighth intercostal space was performed. The herniated liver was placed back in the abdomen, and the defect was closed with interrupted non-absorbable sutures (Figure 2). No mesh was used as direct closure was feasible without tension. The postoperative follow-up was unremarkable, and the patient was discharged on postoperative day (POD) 7. The patient is currently fine with no recurrence at 10 years, clinically, and on thoracic x-rays.

### Case 3

**Figure 3:**
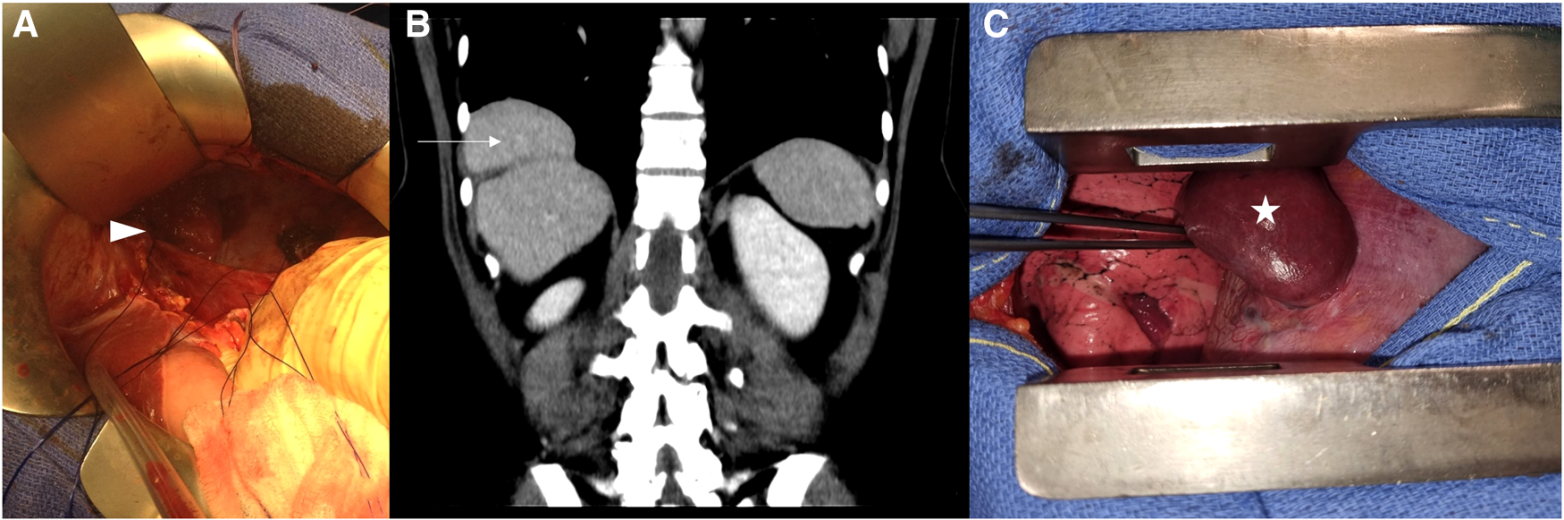
Case 3: (**A**) Upper midline laparotomy after the reduction of the Bochdalek hernia content. Case 4: (**B**) The pre-operative thoraco-abdominal CT in the frontal view is showing the “mushroom” shape of the protruding liver and (**C**) the per-operative view following right thoracotomy, showing the herniated liver before its reduction, presented as a “mushroom” shape.

A 51-year-old female, without a medical history other than three pregnancies, presented with mild dysphagia for a few years, without pyrosis or nausea. An upper endoscopy was evident for gastritis and suspected a hernia process. The CT confirmed the presence of a giant left BH, containing part of the stomach, the left colic angle, the pancreatic tail, and the spleen. Further interrogation did not find recent or former evidence for trauma. Elective surgical management was proposed, but the patient refused. She consulted again 1 year later due to worsening of her symptoms. An abdominal approach was decided, and an upper midline laparotomy was done. Exploration showed no acute strangulation of the protruding organs. After hernial reduction, the defect was huge (Figure 3). The repair required the use of a prosthetic mesh (Gore-Tex®). A Toupet fundoplication was also performed since the His angle was wide open. The postoperative course was uneventful, and the patient was discharged at POD 11. The patient is currently doing fine with no recurrence at 9 years on thoracic imaging.

### Case 4

Our last and most recent case is a 51-year-old female who was diagnosed with an incidental right BH on a CT scan for SARS-CoV-2 infection. Following the interrogation, we did not find evidence for traumatic etiology. Biological liver markers were in the normal range. During her long recovery, chronic coughing aggravated the right thoracic pain. A follow-up CT scan found an increase in the size of the hernia, still with liver content only. The surgical management was postponed twice due to lockdown. The patient was finally prepared for surgery 8 months following her infection, and a right thoracotomy approach was chosen. A “mushroom” aspect of the liver was found herniated in the thoracic cavity (Figure 3). The diaphragmatic defect was enlarged, allowing the reduction of the content. The defect was then closed with a non-continuous non-absorbable suture. The postoperative biological sample showed mild liver cytolysis, but a liver ultrasound ensured the absence of liver necrosis. The patient was discharged at POD 5 and is currently healthy at routine follow-up at 2 years, including annual thoracic x-rays.

## Discussion

**Figure 4:**
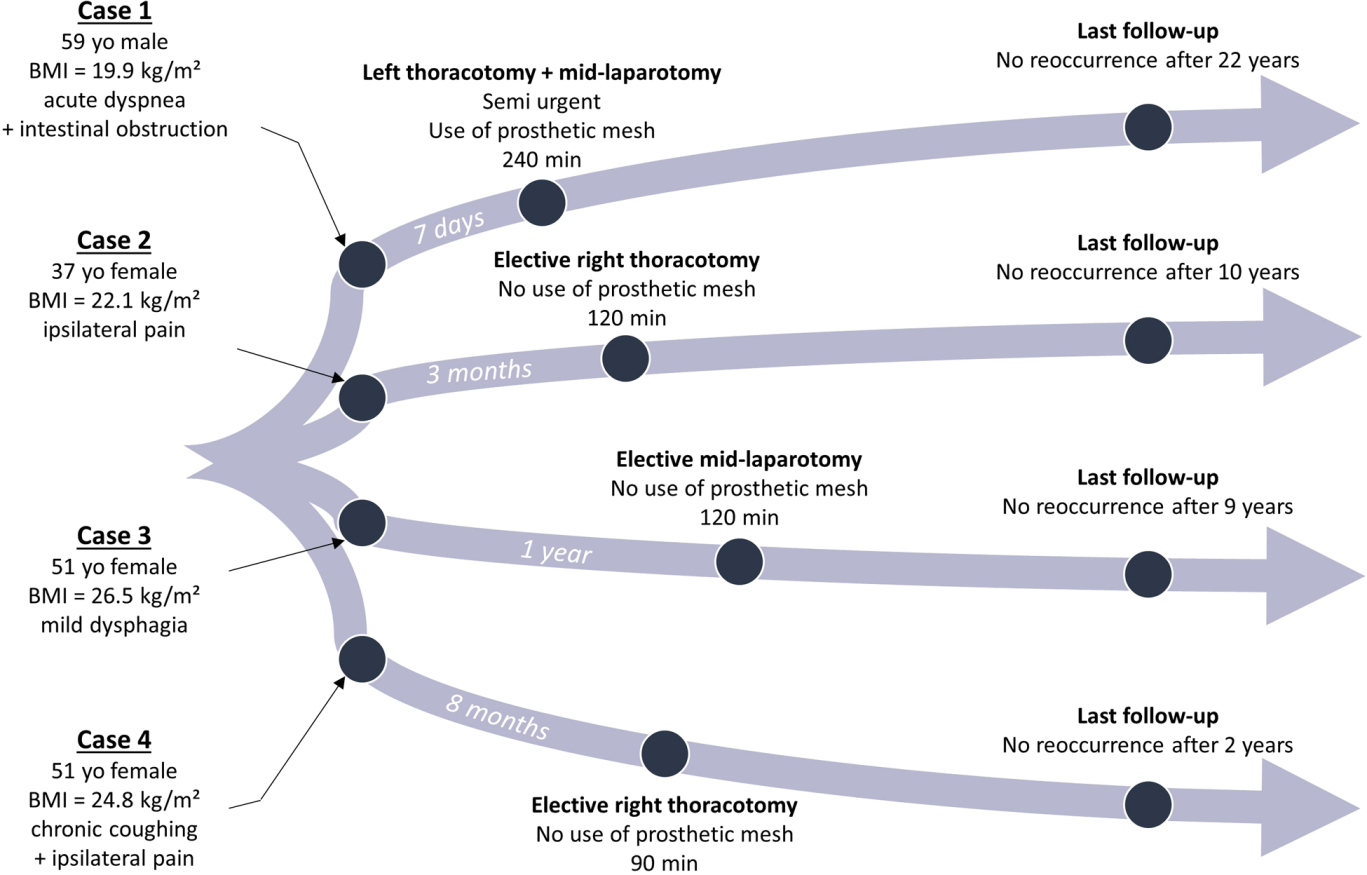
Timeline of each case with clinical characteristics, type of surgical management, and follow-up. BMI, body mass index; yo, years old.

Symptomatic BH among adults remains a rare pathology with nearly a hundred cases published. We report here our experience of four surgical cases and their management at our institution (Figure 4).

The most common profile was identified in a recent literature review and corresponds to a 40-year-old person, of male gender in 55% of cases [3]. The clinical presentation is heterogeneous and has a wide variety of symptoms. Our case series does not derogate from the rule as each patient presented with a different symptomatology, either on the abdominal or the thoracic slope. Brown et al. found that pain, signs of obstruction, and pulmonary symptoms were the three most common symptoms [3]. However, symptoms are not necessarily related to the content of the hernia. Additionally, they appeared in the month preceding the diagnosis in nearly one case out of two. This may bring difficulties for the clinician to diagnose such pathology and can explain why nearly the same amount presents acutely (4). A diversity of organs can be found herniated, but the colon and the stomach are the two most common ones. In some cases, part of the lung can be crushed, and herniated organs can sometimes occupy the entire hemithorax, as in one of our cases.

Left and right BH were equally presented in our series, although left BH is more commonly presented in the literature (70%–90%) (5). This can be explained by two reasons: (i) the closure of the left hemidiaphragm happens later than the right side during the embryological development and (ii) left BH are more often symptomatic than right BH since the liver acts as a fence (6). A causal effect was found in three cases out of the four, although it is usually present in only 25% [3]. Multiple pregnancies are the most frequent causal effect and have been found in two of our cases [3]. Any factor that increases intra-abdominal pressure can aggravate a BH. That is why, a thorough investigation should be held, interrogating the patient for any potential aggravating factors in the past months and even years. Interestingly, we found two precipitating factors, rarely reported if not never: repetitive laparoscopic surgeries in a short time and chronic coughing due to COVID-19. Chronic coughing has been blamed previously for the aggravation of other diaphragmatic defects (7). Knowing that the diagnosis can be suspected clinically or on chest x-ray, a CT scan in most cases can be performed to confirm the diagnosis. A thoraco-abdominal CT will be necessary to evaluate with satisfaction the herniated organs, guiding the management strategy in the presence of suffering signs.

Guidelines on management have not yet been proposed. Nevertheless, the strategy of the management can be distinguished into two axes upon diagnosis: (i) acute complications such as intestinal perforation, abscesses, incarceration with intestinal wall suffering, acute respiratory insufficiency, or hemodynamic instability and (ii) absence of complications, hemodynamic stability, and tolerance of symptoms. In the first case, emergent surgical management must be performed to resolve the life-threatening condition. While in the second case, an elective surgical procedure will be proposed after a full checkup of the patient's condition. The third case can be described as deferred emergent surgery. Indeed, as was the case for our first patient, deferred management offers the possibility to resolve an acute condition such as a non-severe intestinal occlusion or shortness of breath, for instance.

Nowadays, surgical treatment can be performed using different approaches: a traditional open approach, whether by laparotomy or thoracotomy, and minimally invasive approaches, whether laparoscopic, thoracoscopic, or robotic (8). The following criteria will help in the decision-making process: the presence of previous surgeries, the hemodynamic status, the size of the herniated sac, the possibility or not of selective intubation, and the surgeon's experience. Some situations are managed consistently in our department. First, as the diaphragm is at the frontiers of two different specialities, either the lead surgeon is comfortable with both thoracic and abdominal approaches, or both surgical teams should be included in the process. Second, in case of emergent surgical repair, an open approach is usually preferred since bowel resection may be necessary. Third, when the intestine is widely herniated, an abdominal approach may also be more evident, avoiding misidentifying intestinal malrotation (4). For this reason, in our second case, thoracotomy was initially chosen, but an extension to laparotomy and phrenotomy was required. This combined approach is quite frequent, described in 56% of cases in the study of Brown et al. (4). Minimally invasive approaches are currently chosen when feasible, as it decreases the length of hospital stay and the 30-day morbidity rates (4, 9, 10). Furthermore, robotic repair has recently been described as a viable option, improving visualization and ease of intracorporeal suturing (8–11).

The repair of the defect will depend on its size. A direct repair of the defect requires the ability to approximate together both sides. Our strategy is to use a mesh when a direct suture is not possible, notably due to complete or near-complete agenesis. Remarkably, prosthetic repairs are more often performed in a minimally invasive approach than the traditional one (4). Synthetic mesh is usually preferred in elective cases, while biologic ones are reserved for urgent cases requiring bowel resection. In left BH with large defects, a fundoplication might be necessary, reinforcing the subdiaphragmatic level, as was the case in one of our patients. The most common complications following surgery are prolonged gastric ileus and gastroesophageal reflux for left BH, whereas thoracic complications are more common for right BH (4, 12). Finally, the follow-up has only been reported up to 38 months, but our series shows no reoccurrence in 10+ years of follow-up in two cases and 20+ in one case. This underlies the importance of surgical management when BH are symptomatic. The main limitations of this study remain its small number of case reports, its retrospective design, and the absence of minimally invasive management data. However, these are to put in opposition to the very rare nature of such condition. Additionally, the main objective was to bring long-term data on the follow-up, contrasting with the recent data on the feasibility of minimally invasive approaches.

## Conclusion

BH among adults is a rare entity. Its presentation, the content of the hernia, and the aggravating factors are all irregular, bringing difficulties for the diagnosis. Its management should be reserved for specialized centers, with thoracic and digestive teams. Our case series brings clarity to the possibilities of management, depending on the clinical presentation and the organs herniated. It also enlightens the importance of surgical repair when symptomatic, as no reoccurrence has been observed over a period of 10 to 20+ years.

## Data Availability

The raw data supporting the conclusions of this article will be made available by the authors, without undue reservation.
